# Adverse events and predictive probability of peripheral vasopressor administration in pediatric shock: integrating frequentist and hierarchical Bayesian meta-analyses

**DOI:** 10.3389/fped.2025.1719260

**Published:** 2025-11-21

**Authors:** Mario Martinez-Solarte, Jaime Fernández-Sarmiento, Lucía Guzman, Daniel Fernández-Sarta, Lina Gutiérrez-Montenegro, Ana María Sarmiento-Moreno, Hernando Mulett, Maria Carolina Fernández-Palacio, Juan David Suárez, Jonathan Mejía, Raul Copana-Olmos, Nils Casson, Diana Bravo, Anhi Martinez, Daniela Guayambuco, Mariana Ríos, Tejas Girishkumar Mehta, Javier Urbano

**Affiliations:** 1 Department of Pediatrics and Intensive Care, Fundación Cardioinfantil-Instituto de Cardiología, Bogotá, Colombia; 2 School of Medicine and Health Sciences, Universidad del Rosario, Bogotá, Colombia; 3 Department of Pediatrics and Intensive Care, Universidad de La Sabana, Chía, Colombia; 4 Department of Pediatric and Critical Care, Hospital del Niño Manuel Ascencio Villarroel, Department of IIBISMED, Faculty of Medicine, Universidad Mayor de San Simón, Cochabamba, Bolivia; 5 Department of Pediatrics and Intensive Care, Hospital de La Misericordia, Bogotá, Colombia; 6 Department of Pharmacy, Fundación Cardioinfantil—Instituto de Cardiología, Bogotá, Colombia; 7 Department of Pediatrics and Intensive Care, Hamad Medical Corporation, Doha, Qatar; 8 Department of Pediatric Intensive Care, Hospital General Universitario Gregorio Marañón, Maternal and Child Public Health Department, School of Medicine, Complutense University, Madrid, Spain

**Keywords:** vasoactive agents, shock, infusions, catheterization, Bayesian analysis

## Abstract

**Background:**

Pediatric shock demands early vasoactive therapy to restore perfusion. When central access is delayed—particularly in resource-limited settings—peripheral vasopressors provide a feasible alternative. This systematic review and hierarchical Bayesian meta-analysis synthesized current evidence on peripheral vasopressor use in children, estimating incidence, characteristics, and probabilistic risk of local complications to inform safe clinical practice.

**Methods:**

We systematically searched PubMed/MEDLINE, EMBASE, LILACS, Google Scholar, and the Cochrane Library (January 1990–March 2025) using MeSH and free-text terms for vasoactive agents, peripheral intravenous access, pediatric populations, and adverse events. Studies including children aged 1 month–18 years who received peripheral vasopressors and reported local complications were eligible. Two reviewers independently extracted data and assessed study quality using the Joanna Briggs Institute (JBI) checklist. The primary outcome was the pooled proportion of local adverse events. Frequentist and hierarchical Bayesian logistic models estimated pooled rates, 95% confidence or credible intervals, and predictive uncertainty. Weakly informative priors {half-Cauchy for random effects, normal [(0,1)] for log-odds} were applied, and posterior estimates derived via Markov Chain Monte Carlo (four chains, 2,000 iterations, *R^* < 1.01).

**Results:**

Eleven studies comprising 1,575 pediatric patients were included. The pooled incidence of local adverse events was 1.97% (95% CI, 1.41–2.82) with no severe complications such as necrosis, ischemia, or need for surgery. The Bayesian model yielded a consistent pooled rate of 1.8% (CrI95%, 1.0–2.8), with a 68% probability of being below 2% and 99% below 3%. Predictive intervals suggested new studies of 100–300 patients would show rates between 0% and 5%, confirming reproducibility. Subgroup analyses revealed no meaningful differences by setting or catecholamine type. Most events were mild extravasations after a median infusion of 4.1 h (IQR, 2.9–7.1).

**Conclusions:**

Peripheral vasopressor administration in pediatric shock is associated with a very low incidence of local adverse events. The Bayesian hierarchical model confirmed these findings with high probabilistic confidence, supporting peripheral administration as a safe and rapid approach for early hemodynamic stabilization. Peripheral vasopressors may be used for short durations (typically <4 h, according to the included studies) while central access is being established, thereby minimizing delays in critical resuscitation.

**Systematic Review Registration:**

https://www.crd.york.ac.uk/PROSPERO/view/CRD420251115788, identifier CRD420251115788.

## Introduction

Vasoactive agents are a cornerstone in the management of pediatric shock, particularly when fluid resuscitation fails to restore adequate tissue perfusion (1). Traditionally, these medications have been administered through central venous access (CVA) due to concerns about serious local complications, including extravasation, tissue necrosis, and regional ischemia (2, 3). However, establishing CVA in children—especially in emergency settings or resource-limited environments—can be technically demanding, time-consuming, and associated with potentially severe immediate complications (4). In this context, the temporary peripheral administration of vasopressors has emerged as a pragmatic strategy to initiate early hemodynamic support and avoid delays in resuscitation until central access can be safely secured (1, 2).

Current pediatric sepsis guidelines recommend early initiation of vasoactive agents—even via peripheral access—when central access is not readily available, emphasizing the need to prevent delays in effective circulatory restoration (1). Similarly, adult sepsis guidelines recognize this practice as safe and reasonable in urgent scenarios, provided that close monitoring and timely transition to CVA are ensured (5). These recommendations are especially relevant in time-sensitive clinical situations, where delays in vasopressor initiation are consistently linked to worse outcomes (6). Thus, early administration of epinephrine or norepinephrine—regardless of the route—constitutes a critical intervention to achieve hemodynamic stabilization and improve clinical outcomes (1, 5).

Despite its increasing use, the safety profile of peripheral vasopressor infusion in pediatric patients remains a subject of ongoing debate. Recent observational studies have reported low rates of local complications, particularly when appropriate dilutions, short infusion durations, and strict monitoring protocols are applied (7–13). However, heterogeneity in study design, the small number of patients included, the lack of randomized clinical trials directly comparing central versus peripheral vasopressor administration in children with shock, and variability in patient populations, infusion practices, and definitions of adverse events limit the generalizability of these findings. Given these constraints and the rarity of reported adverse events, traditional meta-analytic approaches may provide unstable estimates. Therefore, this systematic review and meta-analysis aimed to synthesize the best available evidence on the use of peripheral vasopressors in children with shock—integrating both frequentist and hierarchical Bayesian frameworks—to more accurately estimate the incidence, characteristics, and probabilistic distribution of local complications.

## Methods

### Search strategy and selection criteria

We conducted a systematic review of major medical databases without language restrictions. The protocol was registered in PROSPERO (PROSPERO ID 1115788. Available at: https://www.crd.york.ac.uk/PROSPERO/view/CRD420251115788). The study was conducted and reported in accordance with the PRISMA guidelines [see Supplementary Digital Content (SDC)].

We included studies that reported adverse events associated with the peripheral administration of vasopressors in pediatric patients (aged 1 month to 18 years) with any type of shock, including septic, cardiogenic, or undifferentiated etiologies. Eligible studies were required to specify the use of peripheral intravenous (PIV) access for vasopressor administration and report at least one related complication, such as extravasation, tissue ischemia, skin necrosis, or limb dysfunction. There were no restrictions on sample size, geographic location, or year of publication (see Supplementary SDC Search strategy).

We excluded studies involving neonates or adult populations, animal models, patients receiving vasopressors exclusively via central venous access, and pharmacokinetic or pharmacodynamic studies lacking safety outcomes. Vasopressor administration through intraosseous access was not considered for analysis, as these devices are mainly used in resuscitation scenarios and, given the lack of procedural standardization across centers, their inclusion could introduce misleading data. Additionally, narrative and systematic reviews, studies that did not report clinical outcomes related to adverse events, and non–peer-reviewed literature—including grey literature (e.g., conference abstracts, OpenGrey, OpenMD), preprints, and unpublished data—were excluded.

### Definitions

For the purposes of this review, peripheral intravenous catheters (PIVs) were defined as devices inserted into and terminating within peripheral veins of the upper or lower extremities (e.g., dorsal hand, antecubital, saphenous), including midline catheters whose tip does not reach the central circulation. Central venous catheters (CVCs) were defined as devices inserted into large proximal veins with the catheter tip terminating in the central circulation, including internal jugular, subclavian, and femoral sites, as well as peripherally inserted central catheters (PICCs). Adverse events were restricted to local anatomic complications directly attributable to peripheral vasopressor infusion (e.g., extravasation, infiltration, local ischemia, skin or soft tissue necrosis), and systemic pharmacologic effects such as tachyarrhythmia, hypertension, or metabolic changes were not considered within the scope of this outcome. We adopted standard terminology as follows: *extravasation* was defined as the unintended instillation of a vesicant medication or solution into the perivascular tissue surrounding the intravenous catheter, whereas *infiltration* referred to the unintended instillation of a non-vesicant medication or solution into the adjacent tissue. These definitions align with those proposed by the Infusion Nurses Society and the Oncology Nursing Society and are widely accepted in pediatric infusion safety protocols (14).

### Data sources and search terms

We systematically searched PubMed/MEDLINE, EMBASE, LILACS, Google Scholar, and the Cochrane Library from January 1990 to March 31, 2025. The search strategy combined the following MeSH and free-text terms: (vasopressor) OR (epinephrine)) OR (norepinephrine)) OR (vasoactive agents)) OR (dopamine)) OR (dobutamine)) OR (vasopressin)) AND (peripheral venous catheter)) OR (peripheral intravenous)) OR (PIV)) OR (infusions)) OR (Catheterization, Peripheral)) AND (complications)) OR (adverse events)) OR (extravasation)) OR (tissue necrosis)) OR (mortality)) OR (infiltration)) AND (children)) NOT (animals)) NOT (case reports)) AND (observational studies)) OR (cohort studies)) OR (cohort)) OR (Clinical trial). The full search strategy is detailed in Supplementary SDC Search strategy.

### Study selection and data extraction

Two reviewers screened titles and abstracts (JFS, MMS), followed by full-text evaluation of potentially eligible studies. Additional collaborators (LGM, DFS, LGM, AMSM, HM, MCFP, JDS, JM, RCO, NC, DB, AM, DG, MR, TG, JU) later joined the review process to assist with data verification, quality appraisal, and manuscript preparation, without modifying the original protocol registered in PROSPERO. Disagreements were first resolved by consensus between HM served as the primary third reviewer for adjudication, and *LGM* acted as alternate adjudicator when required. This hierarchical adjudication process ensured methodological consistency throughout study selection and data extraction. Extracted data included study characteristics (first author, year, country, design), population details (age range, diagnosis, clinical setting), vasopressor type, administration details (peripheral intravenous administration of vasopressors site, catheter size, infusion duration), and the number and nature of adverse events. Patients and/or the public were not involved in the design, conduct, reporting, or dissemination plans of this research.

### Methodological quality assessment

The methodological quality of the included studies was assessed based on both external and internal validity criteria. External validity was evaluated by examining the representativeness of the study population, generalizability of the findings, and control of random error. Internal validity focused on identifying potential sources of bias, including information bias, selection bias, and confounding.

The risk of bias was assessed using the Joanna Briggs Institute (JBI) Critical Appraisal Checklist for Prevalence Studies, which examines nine methodological domains (15). Each item was rated as “Yes”, “No”, “Unclear”, or “Not applicable”. A global judgment of risk of bias was assigned as low (≥7 items met without critical failures), moderate (4–6 items met or one critical failure), or high (≤3 items met or more than one critical failure). The assessment was performed independently by all authors, with discrepancies resolved through discussion and consensus. Full assessments are provided in Supplementary Table S1 and Figure S1*.*

### Outcomes

The primary outcome of this study was to estimate the pooled proportion of adverse events associated with the PIV administration of vasopressors in pediatric patients with any type of shock. Adverse events included, but were not limited to, extravasation, skin or soft tissue necrosis, local ischemia, limb dysfunction, and the need for surgical intervention. Secondary outcomes included the duration of vasopressor infusion via PIV prior to complication or escalation; the type of catheter and anatomical site used; and the dilution or concentration of the vasoactive agents administered peripherally, as reported by the included studies. All outcomes were defined based on the information available in the original reports and were extracted as described by the study authors.

### Data synthesis and statistical analysis

A descriptive analysis was initially conducted to summarize the characteristics of the included studies (Table 1). Proportions (e.g., prevalence of adverse events such as extravasation or tissue injury) were transformed using the Freeman–Tukey double arcsine method to stabilize variance and subsequently meta-analyzed using a random-effects model (DerSimonian–Laird method) with inverse variance weighting. Heterogeneity was evaluated through visual inspection of forest plots and formally quantified using Cochran's *Q* statistic and the *I*^2^ index.

**Table 1:** Characteristics of included studies evaluating peripheral vasopressor administration in pediatric patients with shock.

Author	Year	Country	Age^a^ (IQR)	Study design	Setting	Total PIV (n)	Events (%)	Event type	Septic shock (%)	Trauma (%)	Cardiac arrest (%)	Respiratory disease (%)	Mortality (%)	Bias risk^b^
Abrar et al. (7)	2022	Pakitstan	2.1 (0.5–8.0)	Observational prospective	PICU	369	2.2	Extravasation infiltration	29.0	7.0	10.6	22.0	26.6	9
Charbel et al. (8)	2021	France	0.2 (0.2–12.7)	Observational retrospective	Prehosp. Transport	32	0	None	100.0	N/A	3.1	9.4	ND	8
Copana et al. (16)	2025	Bolivia	1.1 (0.1–17.0)	Observational retrospective	PICU	85	0	None	100.0	N/A	ND	32.9	ND	9
Kohn-Loncarica et al. (9)	2022	Argentina	6.2 (2.0–11.8)	Prospective cohort	ER	56	2.0	Extravasation	100.0	N/A	ND	ND	8.2	7
Kumar et al. (10)	2015	India	ND	Observational retrospective	PICU, ER	204	1.5	Extravasation	66.7	N/A	ND	ND	49.1	9
Lampin et al. (11)	2012	France	2.1 (0.8–6.9)	Observational retrospective	PICU	84	1.2	Extravasation	100.0	N/A	ND	ND	45.2	7
Levy et al. (12)	2022	USA	10.3 (2.6–14.1)	Retrospective cohort	PICU	231	1.7	Extravasation	40.3	6.5	ND	13.0	10.4	7
Mooli et al. (13)	2021	India	ND	Observational retrospective	ER, Ward	84	3.6	Extravasation Tissue necrosis	27.4	31.0	46.4	96.4	ND	9
Patregnani et al. (17)	2017	USA	9.3 (3,8–15.3)	Observational retrospective	PICU, Ward, Prehosp. Transport	102	2.0	Mild edema	61.8	28.4	10.8	ND	16.7	6
Peshiman et al. (18).	2022	United Kingdom	0.25 (0.1–4.1)	Retrospective cohort	Prehosp. Transport	198	3.5	Extravasation	19.7	N/A	4.5	38.4	ND	9
Yeong et al. (19)	2022	Singapore	8.29 (6.2–10.2)	Retrospective cohort	ER	65	0	None	69.2	N/A	9.2	6.2	29.2	9

a Years.

b The risk of bias was assessed using the Joanna Briggs Institute (JBI) Critical Appraisal Checklist for Prevalence Studies Overall risk of bias classification: Low (≥7), Moderate (4–6), High (≤3) (Supplementary Material eTable S1). PIV, Peripheral intravenous route; PICU, Pediatric Intensive Care Unit; ER, Emergency room; IQR, interquartile range; ND, No description; N/A, not applicable.

Subgroup analyses were predefined based on vasopressor type (adrenaline vs. noradrenaline), study design (prospective vs. retrospective) and the methodological quality of the study (high vs. low risk of bias). Sensitivity analyses were performed to evaluate the robustness of the pooled estimates by: (1) applying both fixed- and random-effects models; (2) excluding studies with small sample sizes (<20 participants); and (3) restricting the analysis to higher-quality studies. Consistency of the findings across these analyses was used to support the stability of the results. Publication bias was evaluated through funnel plot asymmetry and tested using the Egger's regression method. A *p*-value < 0.05 was considered statistically significant. All statistical analyses were conducted using R software (version 4.3.1; R Foundation for Statistical Computing, Vienna, Austria) with the packages metafor (Viechtbauer, 2010) and meta (Balduzzi, 2019), as well as Review Manager (RevMan, version 5.4.1; The Cochrane Collaboration, 2020). To ensure methodological coherence and to complement the frequentist synthesis, a Bayesian inferential framework was subsequently applied to the same dataset. This approach was chosen for its ability to estimate the full posterior distribution of the true event rate, directly quantifying the probability of clinically meaningful thresholds rather than relying solely on dichotomous significance testing. The Bayesian model also provides more robust uncertainty estimates in the presence of sparse data and zero-event studies, which are common in pediatric safety research. In this framework, each study contributed evidence to a shared probability structure, allowing the posterior distribution to reflect both within- and between-study variability in adverse event rates. This hierarchical formulation ensured that smaller studies were appropriately weighted while preserving the interpretability of population-level estimates.

### Bayesian hierarchical logistic meta-analysis

To complement the frequentist synthesis, we conducted a hierarchical Bayesian logistic meta-analysis to estimate the population-level rate and between-study variability of local adverse events associated with peripheral vasopressor use in pediatric shock. For each study *i*, the number of events (*yi*) among *ni* patients was modeled as *yi ∼ Binomial*(*ni, pi*), with log*it*(*pi*) *=* μ *+* *ui*, where μ denotes the pooled mean event rate and *ui ∼ Normal*(*0*, τ^2^) captures between-study heterogeneity. Weakly informative priors were chosen (μ ∼ Normal[logit(0.02), 1.5^2^]; τ ∼ Half-Normal[0, 0.5]) to allow broad uncertainty while maintaining realistic constraints on evento probabilities. Sensitivity analyses using broader priors (Normal[logit(0.02), 2.0^2^]; Half-Normal[0, 1.0]) confirmed the robustness of posterior estimates. To ensure robustness in the presence of zero-event studies, we also implemented a hierarchical Beta–Binomial model, where study-specific probabilities followed *pi ∼ Beta(a, b)*, and hyperparameters *(â, b^)* were estimated via empirical Bayes marginal likelihood.

Posterior distributions were derived analytically and through Monte Carlo simulation, yielding 95% credible intervals (CrI 95%) for study-level and pooled estimates. Posterior probabilities for clinically meaningful thresholds—such as *P(event rate* *<* *2%)* and *P(event rate* *<* *3%)*—were computed directly from the posterior density. Posterior predictive checks demonstrated that model-based estimates closely matched the observed data across studies. Predictive intervals were generated for hypothetical future studies of 100–300 patients, and subgroup analyses explored potential moderators (clinical setting, transport environment, and catecholamine combinations). Bayesian computations were performed in Python 3.12 using NumPy, SciPy, and Matplotlib, following reproducible Bayesian inference principles.

## Results

**Figure 1:**
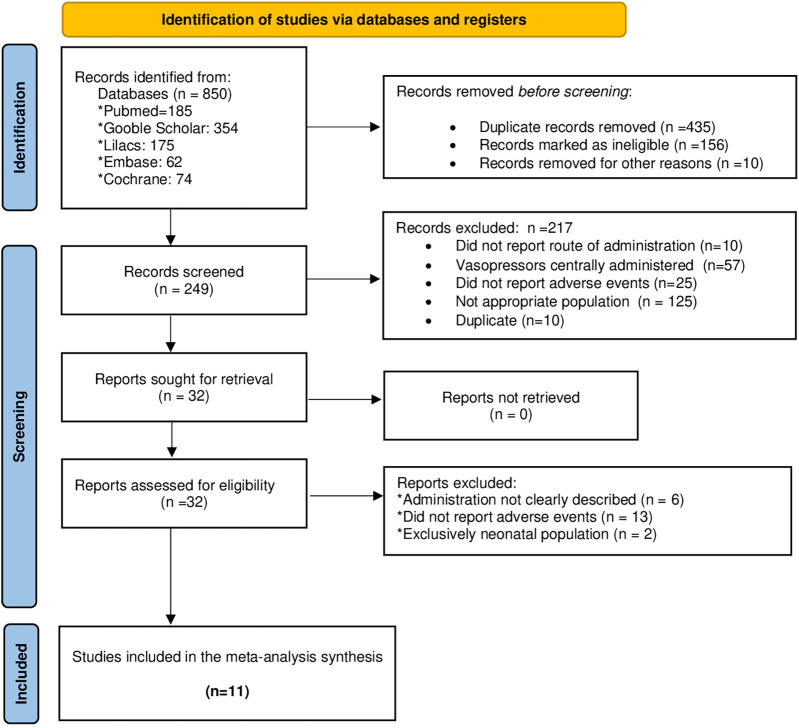
Article selection process according to the PRISMA guidelines for reporting systematic reviews and meta-analyses.

A total of 850 records were identified through database searches (Figure 1). After removing duplicates and screening titles and abstracts, and excluding reviews, animal studies, and articles not meeting eligibility criteria, 11 studies were included in the final analysis (7–13, 16–19). These studies reported data on peripheral administration of vasoactive agents in pediatric patients with shock and described adverse event related to this route of administration.

All included studies had an observational design. No randomized controlled trials were identified that specifically compared peripheral versus central administration of vasopressors in relation to adverse events as the primary outcome. Tables 1, 2 summarizes the main characteristics of the included studies, including country of origin, study design, population, vasopressor type, route and site of administration, and adverse events reported.

**Table 2:** Vasoactive agent administration characteristics in included studies.

Author	Year	Epinephrine n (%)	Epinephrine infusion rate^a^	Norepinephrine n (%)	Norepinephrine infusion rate^a^	Dobutamine n (%)	Dobutamine infusion rate^a^	Dopamine n (%)	Dopamine infusion rate	Combined vasoactive support^b^ (%)	Duration (hours) (IQR)
Abrar et al. (7)	2022	279 (75.6)	0.25	42 (11.4)	0.2	0	0	32 (8.7)	15	20.9	24 (13–1,152)
Charbel et al. (8)	2021	3 (8)	ND	32 (100)	2	7 (19)	ND	0	0	27	3.8 (2.7–4.5)
Copana et al. (16)	2025	20 (23.5)	0.3	51 (60)	0.2	8 (9.4)	ND	6 (7.1)	ND	22.3	ND
Kohn-Loncarica et al. (9)	2022	40 (72)	1	12 (21)	1	3 (5)	6	1 (2)	6	12.5	1 (0.8–2)
Kumar et al. (10)	2015	10 (4.9)	1	0	0	14 (6.9)	20	82 (40.2)	20	98	ND
Lampin et al. (11)	2012	0	0	84 (100)	2.5	ND	18.4	ND	16.5	0	3 (2–4)
Levy et al. (12)	2022	30 (13)	0.1	43 (19)	0.05	0	0	189 (82)^c^	10	48	3,4 (2–8)
Mooli et al. (13)	2021	41 (49)	ND	60 (71)	ND	6	ND	4	ND	ND	12 (1–72)
Patregnani et al. (17)	2017	25 (25)	ND	11 (11)	ND	0	0	85 (87)	ND	48	4.3 (2.4–9)
Peshiman et al. (18)	2022	119 (60.1)	0.2	20 (10.1)	0.5	0	0	88 (44.4)	10	13.6	3 (1.8–5.3)
Yeong et al. (19)	2022	23 (34.8)	0.5	6 (9.1)	0.2	7 (10.6)	30	47 (71.2)	30	24.6	2.4 (1.5–4)

Number and proportion of patients receiving each vasoactive medication, infusion rates, frequency of combined vasoactive support, and infusion duration. Percentages are calculated over the total number of patients receiving peripheral venous infusions. Since some children received more than one vasopressor simultaneously, percentages are based on the total number of infusions; therefore, cumulative percentages may exceed 100%.

a Referred to maximum dose as μg/kg/min units.

b Two or more combinations between vasopressor and inotrope, only vasopressors or only inotropes agents. IQR, interquartile range; ND, no description.

c A total of 31 patients who received more than one infusion were described, accounting for 262 infusions in total.

The methodological quality of the included studies was assessed using the JBI Critical Appraisal Checklist for Studies Reporting Prevalence Data. A total of 11 observational studies were evaluated, of which 10 (90.9%) were judged to have low risk of bias and 1 (9.1%) moderate risk. Most studies demonstrated a representative sampling frame, appropriate participant recruitment, clear case definitions. In the assessment of publication bias, visual inspection of the funnel plot for the 11 included studies revealed a symmetrical distribution of points around the pooled estimate, with no evidence of marked asymmetry. This pattern suggests a low likelihood of publication bias, a finding consistent with the non-significant result of Egger's test (*p* > 0.05) (see Supplementary Figure S2).

### Outcomes

#### Local adverse events associated with peripheral vasopressor use

We identified a total of 1,575 pediatric patients who received vasoactive or inotropic agents PIV access. Dopamine was the most commonly administered agent (37.1%, *n* = 585), followed by epinephrine (35.0%, *n* = 552), norepinephrine (16.8%, *n* = 264), and dobutamine (6.0%, *n* = 95). Based on standardized classification, 8 studies were categorized as retrospective observational (8, 10, 12, 13, 16–19), 2 as prospective cohort (7, 9), and 1 as retrospective multicenter (11). The median age of the population was 25 months (IQR 1–180), and 49.9% were male. The median weight was 10 kg (IQR 7.4–25.3). The median length of stay in the pediatric intensive care unit (PICU) was 7 days (IQR 5.3–8.3).

**Figure 2:**
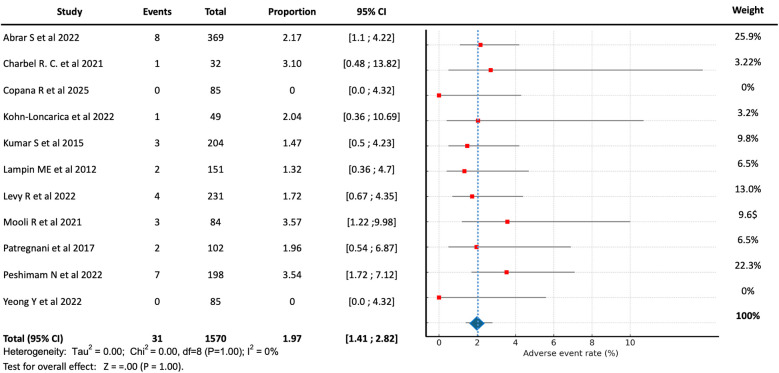
Forest plot showing the pooled proportion and 95% confidence intervals of local adverse events with peripheral vasopressor use in pediatric shock.

Adverse events associated with PIV vasopressor use occurred in 31 children, representing an overall frequency of 1.97% (95% CI, 1.41–2.82) among the exposed population (Figure 2). Most patients received vasopressors in the PICU setting (64.6%), followed by the emergency department (26.9%), inpatient wards (6.2%), and during inter-hospital transport (2.3%). Reported adverse events included mild extravasation (e.g., local erythema or infiltration) and edema without intervention. Only one study reported local tissue necrosis at the infusion site secondary to extravasation, with complete recovery in all cases (13). Notably, no cases were described as requiring surgical debridement, nor were there reports of severe skin burns or mortality attributable to the PIV administration of vasopressors.

### Characteristics of peripheral administration: infusion duration, catheter site, and vasoactive agent concentration

Among the included studies, the most common anatomical sites for PIV vasopressor initiation were the upper extremities (antecubital and dorsal hand veins), although detailed location reporting was inconsistent across studies. Most studies did not specify whether complications occurred during or after infusion discontinuation, nor did they stratify outcomes by drug type.

The duration of vasopressor infusion via PIV access was reported in eight of the included studies. The median infusion time was 4.07 h (IQR 2.85–7.13). The longest recorded infusion was 24 h and was reported by Abrar et al. (7) who administered vasopressors through an antecubital vein. The maximum dose of epinephrine administered via PIV was 0.3 mcg/kg/min (IQR 0.1–0.3), and for norepinephrine, it was 0.6 mcg/kg/min (IQR 0.1–1.2).

Peripheral dilution strategies varied considerably among the studies. For epinephrine, the median dilution was 20 mcg/mL (IQR 6–80), while for norepinephrine it was 16 mcg/mL (IQR 6–80). These concentrations reflect institutional practices rather than unified protocols. Notably, no study reported a correlation between drug dilution and adverse events. Furthermore, most studies failed to differentiate whether specific dilutions were associated with certain catheter types or anatomical sites, highlighting the need for standardization in future research.

### Subgroup analysis

To better isolate the risk associated with guideline-recommended vasoactive agents, we conducted a subgroup analysis excluding patients who received dopamine. Among the 141 pediatric patients who received epinephrine and/or norepinephrine without dopamine, 4 experienced local adverse events, yielding a proportion of 2.84% (95% CI, 1.11–7.08). This analysis suggests a low overall complication rate when these catecholamines are administered peripherally without concurrent dopamine exposure. No significant differences were observed in subgroup analyses stratified by study design (prospective vs. retrospective) or by risk of bias classification (low vs. moderate). Sensitivity analyses, including alternative statistical models, exclusion of small studies, and restriction to higher-quality studies, yielded results consistent with the main analysis, supporting the robustness of the pooled estimates.

### Bayesian results and predictive analysis

The hierarchical Bayesian model, which incorporated study-level variability and uncertainty, yielded estimates consistent with the frequentist analysis, while providing richer probabilistic interpretation of safety thresholds. The hierarchical Beta–Binomial model yielded hyperparameters *â* = 16.48 and *b^* = 889.29, corresponding to a pooled mean event rate of 1.8% (95% CrI 1.0%–2.8%). The posterior probability that the true event rate was below 2% was 68%, and the probability of being below 3% was 99%, strongly supporting the safety of peripheral access as an initial strategy for vasopressor delivery in pediatric shock.

**Figure 3:**
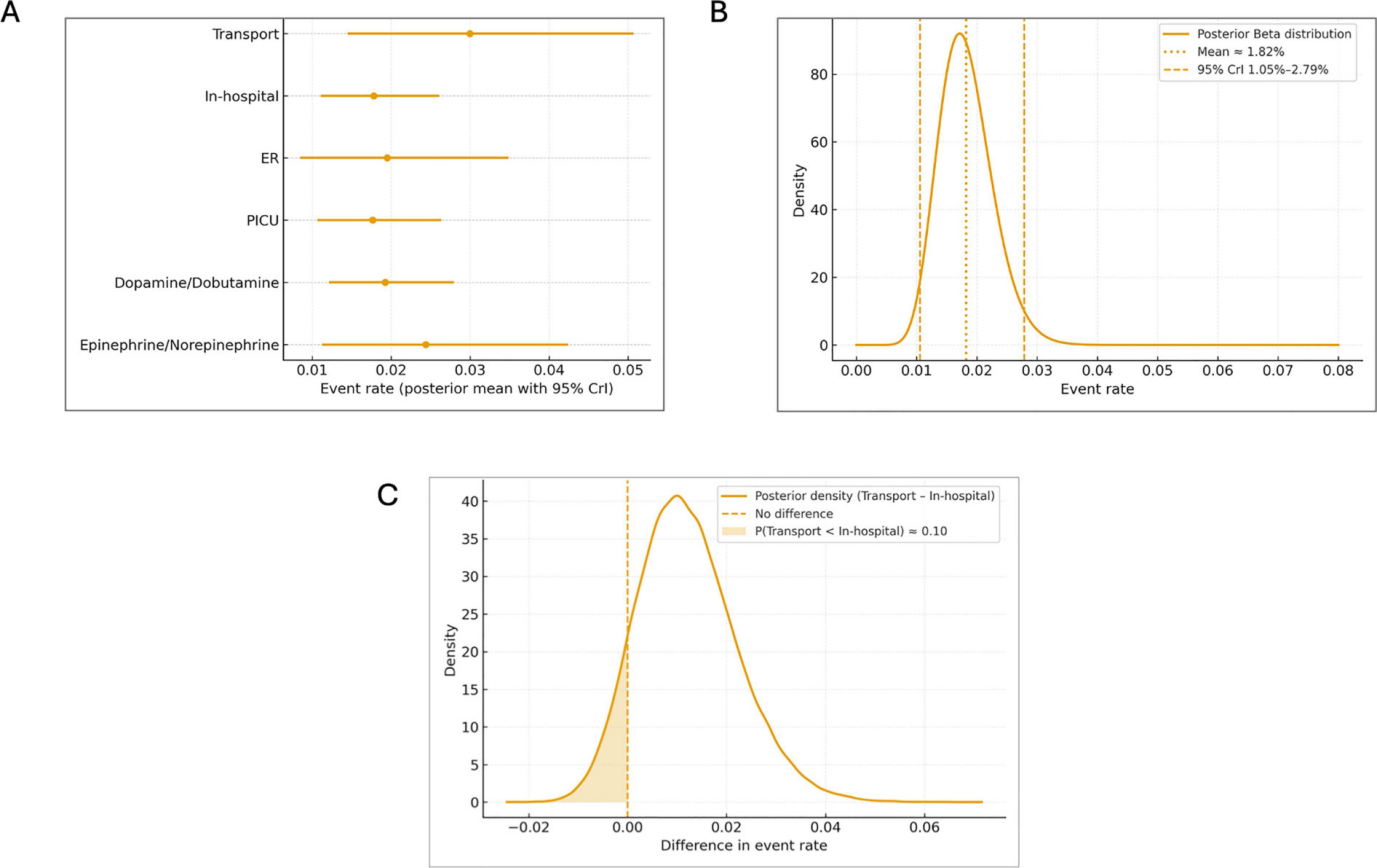
Bayesian hierarchical meta-analysis of peripheral vasopressor safety by subgroups. **(A)** Posterior mean event rates with 95% credible intervals (CrI95%) across key subgroups: transport, in-hospital, ER, PICU, and catecholamine regimen (dopamine/dobutamine vs. epinephrine/norepinephrine). Each dot represents the posterior mean estimate, and horizontal lines denote 95% CrI. The figure illustrates overlapping intervals and the absence of clinically meaningful differences among subgroups. **(B)** Population posterior distribution of event rates (Beta hierarchical model). Posterior Beta distribution representing the estimated population-level rate of local adverse events associated with peripheral vasopressor use. The solid curve depicts the posterior density, with vertical dashed lines marking the 95% credible interval (CrI95%). The dotted red line indicates the posterior mean (1.8%), corresponding to the pooled hierarchical estimate. The narrow interval reflects high precision and consistency across included studies. **(C)** Posterior difference in event rates (Transport vs. In-hospital). Posterior density of the difference in event rates between transport and in-hospital settings. The shaded blue area represents the posterior probability that the transport subgroup has a lower event rate (P[Transport < In-hospital] ≈ 0.93). The dashed vertical line marks the null difference (0). The posterior distribution supports the inference of minimal and clinically non-relevant differences between environments.

Bayesian predictive intervals indicated that in a new study of 100–300 patients, the expected complication rate would consistently fall between 0% and 4%–5%, reinforcing the reproducibility and stability of this finding across clinical contexts. The Bayesian meta-regression by subgroups (clinical setting, transport, and use of combined catecholamines) did not reveal clinically relevant differences. Figure 3A illustrates the posterior Beta distribution of event rates, whereas Figure 3B presents subgroup estimates with overlapping credible intervals, and Figure 3C demonstrates the minimal posterior difference between transport and in-hospital settings.

However, the transport subgroup demonstrated a mean rate of 3.0% (95% CrI 1.5%–5.1%), with a 93% probability of being slightly higher than in-hospital studies (1.8%, 95% CrI 1.1%–2.6%), possibly reflecting logistical and technical factors inherent to prehospital care. Rates were similar across ER (2.0%; 95% CrI 0.9%–3.5%) and PICU (1.8%, 95% CrI 1.1%–2.6%) environments. No meaningful difference was observed between studies including dopamine/dobutamine combinations (1.9%, 95% CrI 1.2%–2.8%) and those using epinephrine/norepinephrine alone (2.0%, 95% CrI 1.3%–2.7%). The Bayesian posterior distribution therefore allows clinicians to interpret safety in probabilistic terms—indicating, for instance, that in more than 99 of 100 comparable future scenarios, the true event rate would remain below 3%, reinforcing the clinical reproducibility of these findings.

## Discussion

In this systematic review and meta-analysis, we found that the proportion of local adverse events associated with the temporary peripheral administration of vasoactive agents in pediatric patients with shock is low, with an overall incidence of 1.97%. Importantly, no cases of severe complications were reported across the included studies. None of the adverse events required surgical intervention, nor were there reports of permanent sequelae or functional impairment of the affected extremity. These findings support the use of peripheral vascular access for the initial administration of vasoactive agents as a pragmatic strategy to prevent delays in hemodynamic stabilization, particularly in limited-resource settings where central venous catheter (CVC) placement may not be immediately feasible. This approach is particularly valuable when peripheral administration is used as a time-limited intervention, accompanied by a structured plan for transition to central access once the patient's condition allows. It becomes especially relevant in situations where central venous catheter (CVC) placement may be delayed—such as during transport, in emergency departments, or in resource-constrained environments—where immediate vascular access is critical. In these contexts, the short-term use of peripheral vasopressors while central access is being secured represents a pragmatic, safe, and time-sensitive strategy to prevent delays in hemodynamic stabilization.

Although vasopressors have traditionally been administered via central venous access due to concerns regarding extravasation and local tissue injury, recent evidence supports the safety and feasibility of short-term peripheral administration, particularly when central access is delayed or not readily achievable. In adults with septic shock, peripheral infusion—preferably through a catheter placed proximal to the antecubital fossa and for a duration of less than six hours—has been associated with a low incidence of extravasation (3.4%), with no reported cases of tissue necrosis or limb ischemia and rarely necessitates any active intervention (2). While randomized trial data remain limited, observational studies and meta-analyses suggest that a substantial proportion of patients can be managed without requiring central venous catheterization, and that the time to vasopressor initiation is significantly reduced when peripheral access is utilized (6).

The rationale for early peripheral vasopressor initiation lies in its capacity to mitigate the deleterious cascade of persistent hypotension, including prolonged tissue hypoperfusion, fluid overload, and exacerbation of capillary leak syndrome (20–24). Evidence from Copana et al. multicenter study in a resource-constrained setting demonstrated a significant reduction in mortality (adjusted OR 0.49; 95% CI 0.28–0.89) when vasoactive agents were initiated within the first hour of persistent hypotension, predominantly via peripheral routes (16). These findings align with the pathophysiological principles emphasized in recent consensus recommendations, which advocate for early vasopressor support to counter systemic vasodilation and avoid fluid resuscitation strategies that may perpetuate endothelial injury and worsen clinical outcomes (1, 5). This is particularly relevant in limited-resource settings, where trained personnel for central venous catheter insertion are not always available, leading to potential delays in initiating life-saving vasoactive therapy.

Our findings are consistent with previous observational studies that reported similarly low rates of adverse events with peripheral vasopressor administration. Abrar et al. described a 2.2% incidence of extravasation events in a cohort of critically ill children, none of which progressed to tissue injury (7). Takeshita et al. comprehensive meta-analysis, which included both peripheral and intraosseous access, corroborated these results, reporting a pooled incidence of 2.3% for local adverse events, thus reinforcing that central access is not an absolute prerequisite for the safe administration of vasoactive agents (25).

Despite the low incidence of adverse local events, available evidence is highly heterogeneous across patient age, clinical settings, and study design. Pediatric populations spanned from infants to adolescents, with wide variation in infusion protocols, monitoring capacity, and thresholds for transitioning to central venous access. A major gap is the absence of consensus on the maximum vasoactive concentration that can be safely infused through a given caliber of peripheral vein. Reported practices often reflected institutional routine rather than evidence-based thresholds, and few studies stratified complication rates by drug dilution, catheter size, or insertion site. This variability and incomplete reporting hinder the development of universal recommendations for concentration–caliber combinations in children. While this analysis supports the low frequency of adverse events when peripheral vasopressors are administered temporarily in pediatric shock, individualized judgment—guided by local protocols—remains essential until prospective studies clarify optimal drug concentration, catheter size, anatomical site, and patient age.

The variability in vasopressor dilutions across studies highlights a critical knowledge gap regarding concentration-dependent risk. Reported concentrations ranged from 6 to 80 µg/mL for both epinephrine and norepinephrine, with most institutions using intermediate dilutions around 16–20 µg/mL. Notably, none of the included studies demonstrated a linear relationship between drug concentration and the occurrence of extravasation or tissue injury, suggesting that infusion duration, catheter location, and vessel caliber may be more influential determinants of local safety. This heterogeneity precluded a formal meta-regression analysis, but the hierarchical Bayesian model—by accounting for uncertainty and study-level variability—showed a consistently low posterior probability (<3%) of adverse local events across all concentration ranges. These findings support that within commonly used clinical dilutions and short infusion periods (<4 h), peripheral vasopressor administration remains a safe and reproducible practice.

The hierarchical Bayesian framework provided a powerful and clinically intuitive method to quantify the safety profile of peripheral vasopressor use in children. Unlike traditional frequentist analyses, which rely on *p*-values, the Bayesian approach yields direct probability estimates of clinically meaningful outcomes—allowing statements such as a *99% probability that the true event rate is below 3%*. This probability-based interpretation aligns better with clinical reasoning and decision-making in time-sensitive critical care settings.

Our findings confirm that peripheral administration of epinephrine or norepinephrine in pediatric shock is a safe, pragmatic, and low-risk strategy, consistent across diverse care settings and patient populations (26). The narrow dispersion of posterior estimates and overlapping credible intervals underscore the robustness of this conclusion (27). From a methodological standpoint, the hierarchical Bayesian approach is particularly suited for pediatric critical care research, where studies often have small sample sizes and low event rates (28, 29).

The reproducibly low incidence of adverse events likely reflects a combination of modifiable procedural and physiologic factors. Across studies, vasopressors were infused for short durations (median 4 h, IQR 2.9–7.1) through upper-extremity veins—most commonly antecubital or proximal forearm sites—using diluted concentrations within the 16–20 µg/mL range and under strict visual monitoring. These strategies reduce extravasation pressure gradients and limit local *α*-adrenergic vasoconstriction, preserving tissue perfusion. Importantly, such structured protocols demonstrate that the safety of peripheral vasopressor administration is not incidental but the result of consistent adherence to best practices that can be feasibly replicated in routine clinical care, particularly when central access is delayed.

This recommendation is particularly relevant in resource-limited settings, where trained personnel for central line placement are not always available in emergency or transport environments. In such circumstances, delays in initiating vasoactive therapy in sepsis have been consistently associated with worse outcomes. The Bayesian analysis reinforces this clinical rationale by providing a high-probability estimate of safety, supporting peripheral vasopressor use as a pragmatic, timely, and evidence-based strategy for early hemodynamic optimization.

### Limitations

We acknowledge several limitations in this study. All included studies were observational, as no randomized clinical trials have specifically compared the efficacy, safety, or incidence of adverse events between peripheral and central vascular access for vasopressor administration in children with shock. Consequently, the observational design inherently limits causal inference due to potential residual confounding and bias. However, as recent methodological advancements emphasize, well-designed observational studies, when embedded within explicit causal frameworks and grounded in robust assumptions, can yield valid causal interpretations, particularly in clinical scenarios where randomized controlled trials are ethically or logistically unfeasible (30). Furthermore, heterogeneity across studies regarding patient populations, catheter insertion sites, monitoring protocols, dilution strategies, and definitions of adverse events introduces clinical and methodological variability that may impact the generalizability of our findings. Although we sought to mitigate this through strict inclusion criteria and subgroup analyses, residual heterogeneity remains a limitation. Additionally, the lack of standardized reporting on adverse event severity and management poses challenges in accurately determining the true incidence and clinical relevance of complications associated with peripheral vasopressor administration. Underreporting of adverse outcomes cannot be entirely excluded, potentially leading to publication bias favoring studies with more favorable safety profiles. Another limitation is that time-to-extravasation data were not systematically reported in the primary studies, preventing an accurate estimation of the temporal relationship between infusion duration and the onset of local adverse events.

## Conclusion

Our systematic review and meta-analysis, integrating both frequentist and hierarchical Bayesian approaches, found that peripheral administration of vasoactive agents in pediatric shock is associated with a very low incidence of local adverse events and no severe complications. The Bayesian model confirmed these findings with high probabilistic confidence, reinforcing the safety and reproducibility of this practice across diverse clinical settings. These results support the early and time-limited (<4 h) use of peripheral vasopressors while central venous access is being established, particularly in scenarios where delays in central line placement could worsen patient outcomes. The implementation of structured institutional protocols—focused on catheter site selection, drug dilution, and standardized monitoring—will be essential to ensure safe and effective adoption of this strategy in routine pediatric critical care.

## Data Availability

The raw data supporting the conclusions of this article will be made available by the authors, without undue reservation.
