# Supplementary material for: Adverse events and predictive probability of peripheral vasopressor administration in pediatric shock: integrating frequentist and hierarchical Bayesian meta-analyses
Source: Front Pediatr. 2025 Nov 21;13:1719260. doi: 10.3389/fped.2025.1719260 (PMC12678326; doi:10.3389/fped.2025.1719260)

**Adverse Events and Predictive Probability of Peripheral Vasopressor Administration in Pediatric Shock: Integrating Frequentist and Hierarchical Bayesian Meta-Analyses**

Mario Martinez-Solarte MD^1,2^, Jaime Fernández-Sarmiento MD, PhD^1,3^, Lucía Guzman MD^1,3^, Daniel Fernández-Sarta^1^, Lina Gutiérrez-Montenegro MD^1,3^,  Ana María Sarmiento-Moreno MD^1,^ Hernando Mulett MD^1,2^, Maria Carolina Fernández-Palacio MD^1,3^, Jonathan Mejía MD ^1,2^, Raul Copacana MD MSc^4^ , Nils Casson MD^5^, Diana Bravo MD^6^, Anhi Martinez RPh^7^, Tejas Girishkumar Mehta MD^8^, Javier Urbano MD PhD^9^

^1^ Department of Pediatrics and Intensive Care. Fundación Cardioinfantil-Instituto de Cardiología. Bogotá, Colombia.

^2^ School of Medicine and Health Sciences. Universidad del Rosario. Bogotá, Colombia.

^3^ Department of Pediatrics and Intensive Care. Universidad de La Sabana. Chía, Colombia.

^4^ Department of Pediatric and Critical Care, Hospital del Niño Manuel Ascencio Villarroel, Department of IIBISMED, Faculty of Medicine, Universidad Mayor de San Simón, Cochabamba, Bolivia.

^5^ Division of Pediatric Intensive Care Unit, Hospital San Juan de Dios. Tarija, Bolivia.

^6^ Department of Pediatrics and Intensive Care. Hospital de La Misericordia. Bogotá, Colombia.

^7^ Department of Pharmacy, Fundación Cardioinfantil – Instituto de Cardiología, Bogotá, Colombia.

^8^ Department of Pediatrics and intensive Care. Hamad Medical Corporation. Doha, Qatar.

^9^ Department of Pediatric Intensive Care, Hospital General Universitario Gregorio Marañón. Maternal and Child Public Health Department, School of Medicine, Complutense University, Madrid, España.

**Supplemental Digital Content**

**Corresponding autor:**

Jaime Fernández-Sarmiento MD, PhD

E-mail: jaimefe@unisabana.edu.co

**Table of contents**

1. **Search Strategy** (PICO Framework) Pag 4
2. **eTable1:** Risk of Bias Assessment Using the JBI Critical Appraisal Checklist for Studies Reporting Prevalence Data Pag 5
3. **Figure S1.** Risk of Bias Assessment Using the JBI Critical Appraisal Checklist for Studies Reporting Prevalence Data. Pag 6
4. **Figure S2**. Funnel plot of included studies evaluating local adverse events with peripheral vasopressor administration in pediatric patients with shock. Pag 7
5. Preferred Reporting Items for Systematic Reviews and Meta-Analyses (PRISMA) Checklist Pag 8,9
6. **Search Strategy (PICO Framework)**

The following table outlines the PubMed search strategy developed for this systematic review, structured according to the PICO framework. Medical Subject Headings (MeSH) and free-text terms were combined to capture relevant studies, with filters applied to exclude animal-only studies and case reports.

1. **P (Population / Intervention):**

"Vasoconstrictor Agents"[MeSH] OR "Epinephrine"[MeSH] OR "Norepinephrine"[MeSH] OR "Dopamine"[MeSH] OR "Vasopressin"[MeSH] OR vasopressor*[tiab] OR vasoactive[tiab] and children OR child*

1. **I (Route of Administration):**

"Infusions, Intravenous"[MeSH] OR "Catheterization, Peripheral"[MeSH] OR peripheral intravenous[tiab] OR PIV[tiab]

1. **Study Design Criteria (cohort, quasi-experimental, RCT):**

"Cohort Studies"[MeSH] OR cohort*[tiab] OR "Controlled Clinical Trial"[Publication Type] OR randomized controlled trial[pt] OR quasi-experimental[tiab] OR "Controlled Before-After Studies"[MeSH]

1. **Limits / Exclusions:**

NOT animals[mh] NOT case reports[pt]

 (Search: ((((((((((((((((((((((vasopressor) OR (epinephrine)) OR (norepinephrine)) OR (vasoactive agents)) OR (dopamine)) OR (dobutamine)) OR (vasopresin)) AND (peripheral venous catheter)) OR (peripheral intravenous)) OR (PIV)) OR (infusions)) OR (Catheterization, Peripheral)) AND (complications)) OR (adverse events)) OR (extravasation)) OR (tissue necrosis)) OR (mortality)) OR (infiltration)) AND (children)) NOT (animals)) NOT (experimental study)) NOT (case reports))

1. **eTable 1 .**  **Risk of Bias Assessment Using the JBI Critical Appraisal Checklist for Studies Reporting Prevalence Data**

**Item descriptions:**

**Item 1:** Was the sample frame appropriate to address the target population?

**Item 2:** Were study participants sampled in an appropriate way?

**Item 3:** Was the sample size adequate?

**Item 4:** Were the study subjects and the setting described in detail?

**Item 5:** Was the data analysis conducted with sufficient coverage of the identified sample?

**Item 6:** Were valid methods used for the identification of the condition?

**Item 7:** Was the condition measured in a standard, reliable way for all participants?

**Item 8:** Was there appropriate statistical analysis?

**Item 9:** Was the response rate adequate, and if not, was the low response rate managed appropriately?

Y = criterion met; N = criterion not met; U = unclear; NA = not applicable.

Overall risk of bias classification: Low (≥7 “Yes” responses without critical flaws), Moderate (4–6 “Yes” responses or one critical flaw), High (≤3 “Yes” responses or multiple critical flaws).

1. **Figure S1. Risk of Bias Assessment Using the JBI Critical Appraisal Checklist for Studies Reporting Prevalence Data.**

**
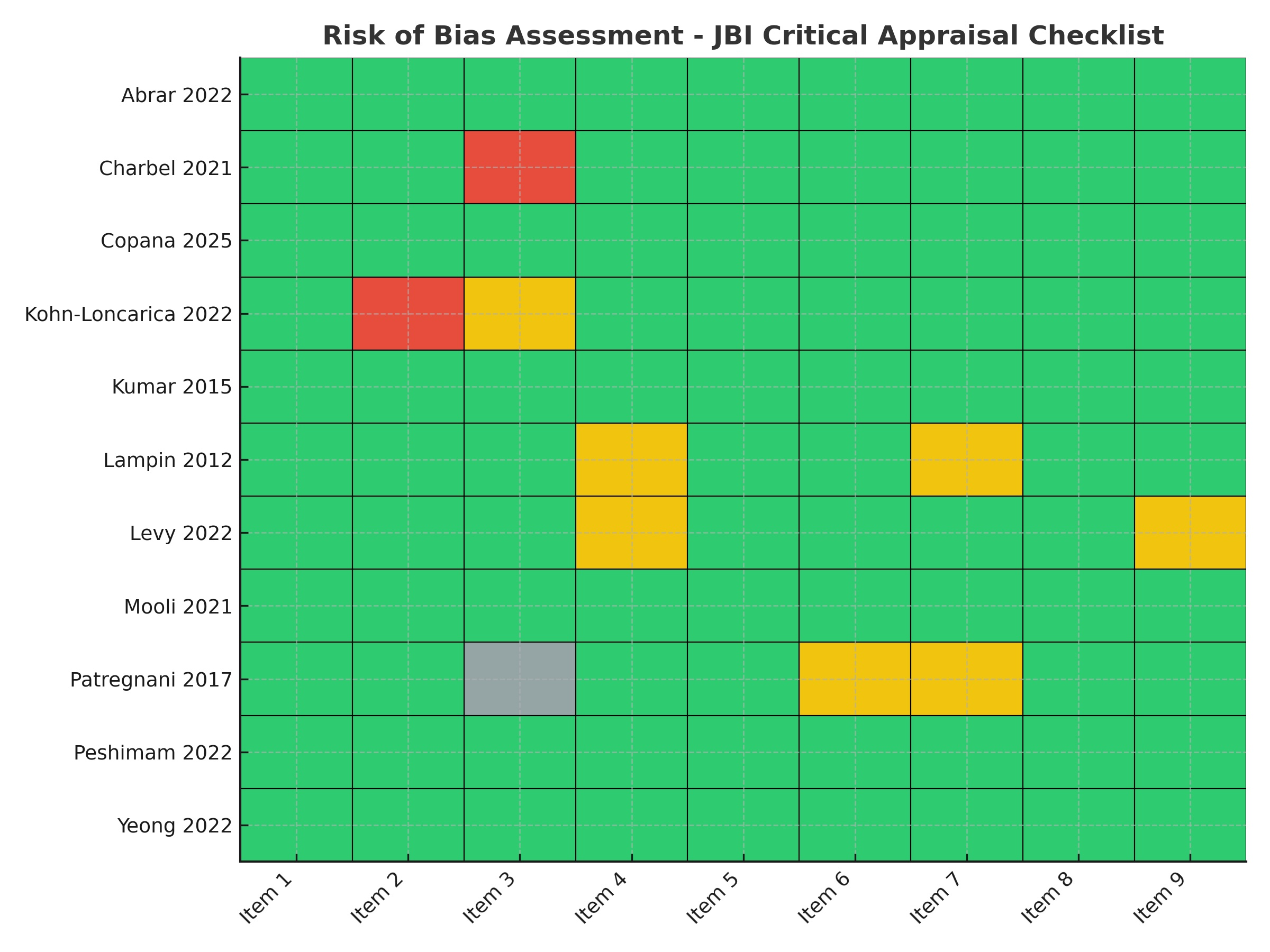
**

Traffic light plot illustrating the risk of bias assessment for each included study across the nine JBI domains: 1, appropriate sample frame; 2, appropriate sampling method; 3, adequate sample size; 4, detailed description of subjects and setting; 5, sufficient coverage of the sample; 6, valid methods for condition identification; 7, standardized and reliable measurement; 8, appropriate statistical analysis; 9, adequate response rate or appropriate management of low response rate. Green = “Yes” (criterion met), red = “No” (criterion not met), yellow = “Unclear”, grey = “Not applicable”. Overall risk of bias classification (JBI Critical Appraisal): Low = ≥7 “Yes” without critical flaws; Moderate = 4–6 “Yes” or one critical flaw; High = ≤3 “Yes” or multiple critical flaws.

1. **Figure S2**. Funnel plot of included studies evaluating local adverse events with peripheral vasopressor administration in pediatric patients with shock


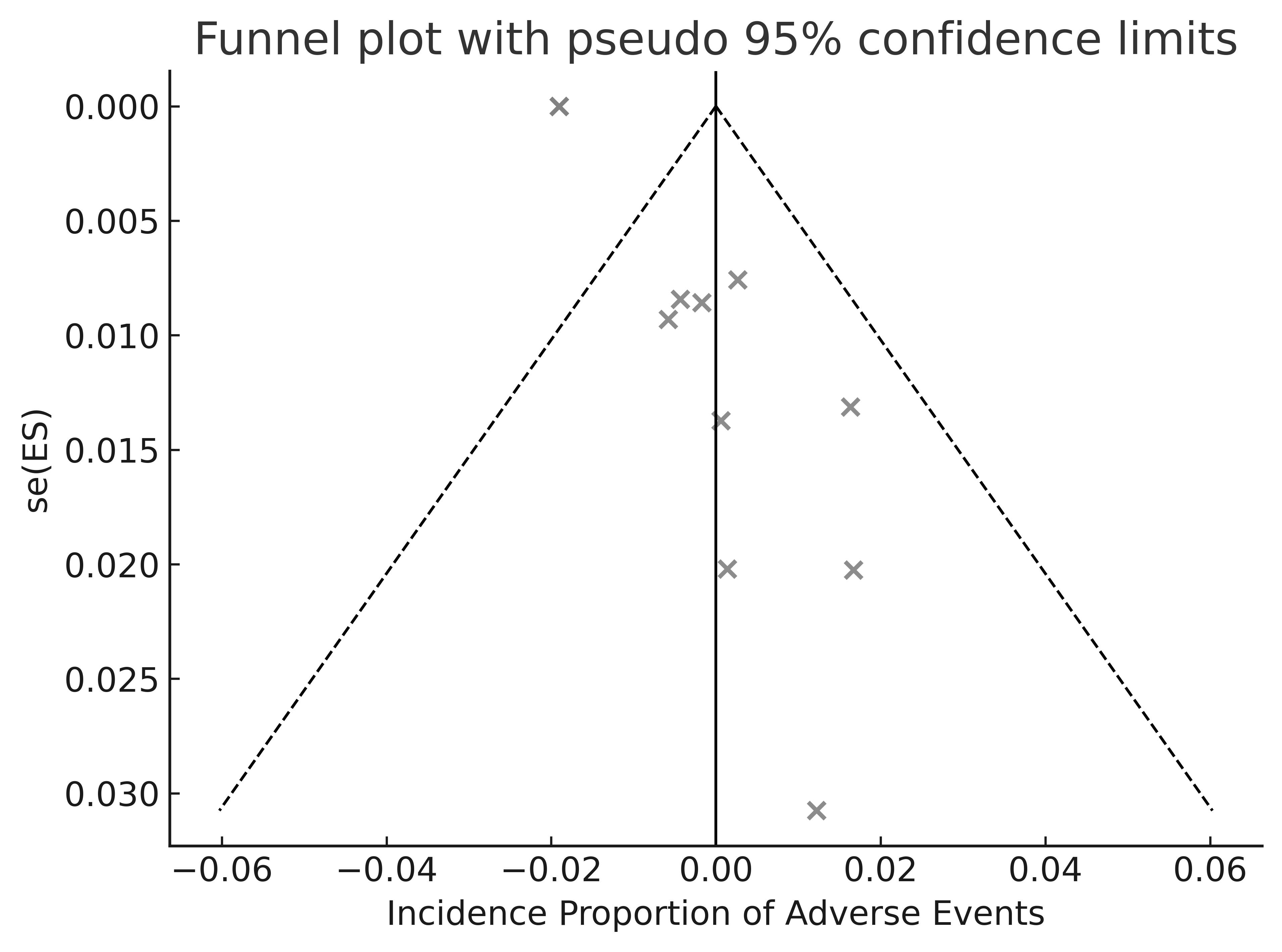


The funnel plot displays the incidence proportion of local adverse events (x-axis) against the standard error of the estimate (y-axis) for the 11 studies included in the meta-analysis. Each point represents an individual study. The vertical solid line indicates the pooled proportion, and the dashed lines represent the pseudo 95% confidence limits. Visual inspection shows a symmetrical distribution of studies, suggesting a low likelihood of publication bias

**Preferred Reporting Items for Systematic Reviews and Meta-Analyses (PRISMA) Checklist**


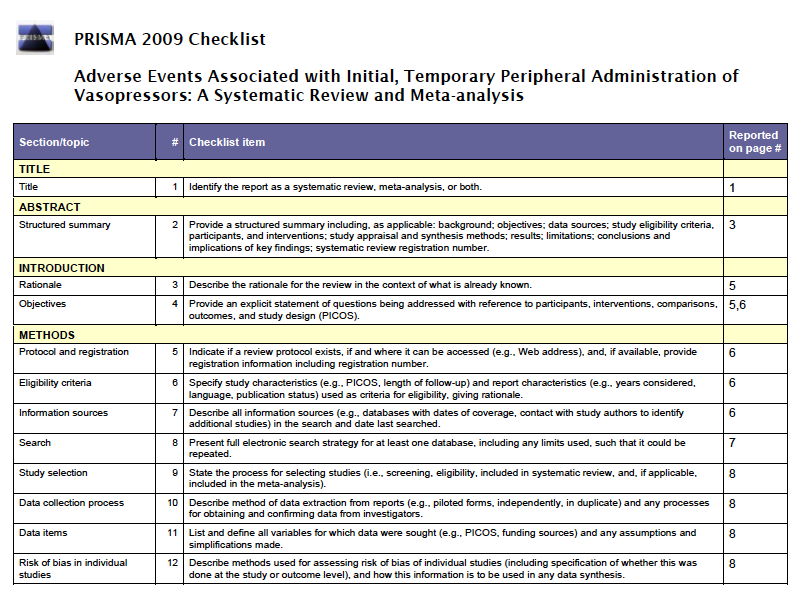


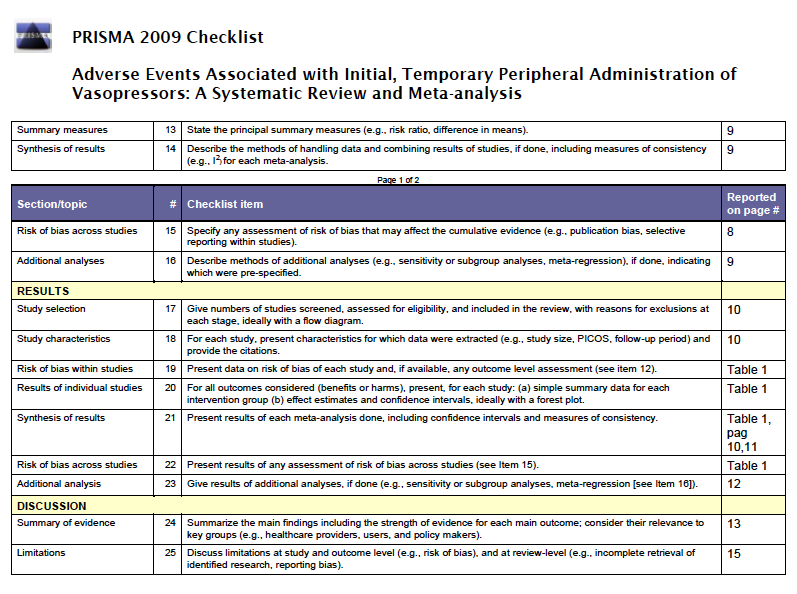


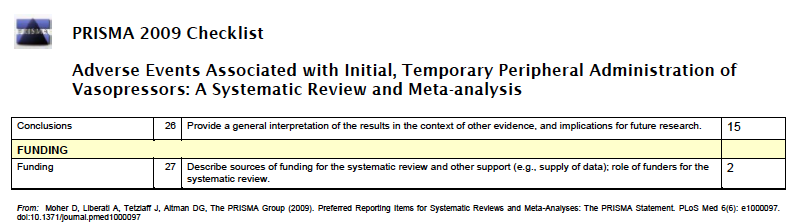

Supplement: Supplementary file 1 [file Supplementaryfile1.docx]
